# Effects of Total Knee Arthroplasty on Coronal and Sagittal Whole-Body Alignments: Serial Assessments Using Whole-Body EOS

**DOI:** 10.3390/jcm10153242

**Published:** 2021-07-23

**Authors:** Seong-Chan Kim, Han-Gyeol Choi, Joo-Sung Kim, Tae-Woo Kim, Yong-Seuk Lee

**Affiliations:** 1 Department of Orthopaedic Surgery, Seoul National University College of Medicine, Seoul National University Bundang Hospital, 166 Gumi-ro, Bundang-gu, Seongnam-si 463-707, Korea; circlein@naver.com (S.-C.K.); meinmed87@naver.com (H.-G.C.); mdndkfc@gmail.com (J.-S.K.); 2 Department of Orthopedic Surgery, Jeju Hyein Foundation Hankook General Hospital, Jeju-si 63183, Korea; 3 Department of Orthopedic Surgery, Seoul National University College of Medicine, Seoul National University Boramae Medical Center, Seoul 07061, Korea; orthopassion@naver.com

**Keywords:** knee, total knee arthroplasty, EOS, coronal alignment, sagittal alignment, spinopelvic alignment, flexion contracture

## Abstract

Background: The aims of this study were to evaluate the effects of correcting lower limb alignment by total knee arthroplasty (TKA) on the spinopelvic alignment and to identify patients with difference in the knee joint between clinically measured passive motion and the actual standing posture. Methods: In this retrospective study, 101 patients who underwent TKA and whose serial whole-body EOS X-ray were available were included. The relationship of the knee and spinopelvic alignment was analyzed by evaluating the parameters of standing anterior-posterior and lateral whole-body EOS X-ray. The differences between postoperative passive motion and weight-bearing posture in the knee joint were assessed in both coronal and sagittal planes. Furthermore, the causes of such differences were analyzed. Results: Significant correlations between Δpelvic obliquity and coronal ΔHip-Knee-Ankle (HKA)_Rt-Lt_ angle between the preoperative and 3-month and 1-year postoperative data (*p* < 0.001 and *p* < 0.005, respectively) and improved with coronal lower limb alignment close to neutral resulted in decreased pelvic obliquity (*p* < 0.001, ß = 0.085 and *p* = 0.005, ß = 0.065, respectively) were observed. The correlations between Δpelvic tilt (PT) and Δsacral slope (SS) and sagittal ΔHKA_Rt-Lt_ angle were statistically significant (PT: *p* < 0.001 and *p* < 0.045; SS: *p* = 0.002 and *p* < 0.001, respectively). The improved sagittal alignment close to neutral resulted in decreased PT and increased SS. The difference between postoperative passive motion and the weight-bearing posture of the knee joint was correlated with lumbar lordosis and sagittal C7 plumb line-sacrum distance (*p* = 0.042 and *p* < 0.001, respectively). Conclusions: The correction of lower limb alignment with TKA affected pelvic parameters dominantly; however, there was little effect on the spinal alignment. Additionally, patients with anterior stooping or lumbar flat back demonstrated difference in extension between passive knee motion and standing. Therefore, rather than only focusing on changes in the knee alignment correction, knee surgeons should also evaluate the spinopelvic alignment before surgery to consider the prognosis of the standing and predict the possible changes in the whole-body alignment. This preoperative assessment may improve the prognosis of TKA.

## 1. Background

Total knee arthroplasty (TKA) is a well-established surgical procedure for correcting deformed painful knees, especially those with varus and flexed deformities, and establishing neutral mechanical alignment [1,2,3,4]. With an increasing aging population, it is common to encounter patients of knee arthritis with concomitant spinal problems because they are the most commonly affected areas due to degenerative changes. Additionally, the spinopelvic alignment can affâect the limb alignment and vice versa [5,6]. This may be related to the compensatory mechanisms that help in maintaining the balance of the whole body. Previous studies have reported some changes in limb alignment following corrections of the spinopelvic alignment [7,8]. Reduction of lumbar lordosis (LL) initially reduces thoracic kyphosis; subsequently, compensatory changes occur in the adjacent peripheral joints to prevent stooping forward and minimize energy consumption in maintaining the line of gravity in the center while standing, resulting in increased pelvic tilt and knee flexion [9,10,11].

However, relatively little is known about the changes in the spinopelvic alignment following correction of the lower limb alignment to improve the balance. Additionally, even after the correction, the posture of the lower limb to compensate for the spinopelvic alignment under actual weight-bearing situations is unclear. We frequently encounter patients who have normal passive knee motions after TKA but walk with a flexed knee, which was assumed to be a compensatory mechanism for balance. This implies that alignment correction is complex because it includes three-dimensional (3D) changes and that it may be related to spinopelvic and lower limb alignments. Predicting the complex changes following correction of the limb alignment with TKA is necessary since some patients can be more uncomfortable during standing or walking if limb alignment correction is performed without considering the spinopelvic alignment. 

Therefore, the purposes of this study were to: (1) evaluate the effects of correcting lower limb alignment with TKA on the spinopelvic alignment and (2) identify the patients who demonstrate differences between clinically measured passive motion and actual standing posture in the knee joint. The hypotheses of this study were that (1) correction of the lower limb alignment with TKA will affect the spinopelvic alignment via compensatory mechanisms, and (2) difference between passive and weight-bearing knee parameters will predominantly manifest in patients with fixed spinopelvic deformities.

## 2. Methods

### 2.1. Demographics

This study retrospectively reviewed the datas of 116 patients who underwent primary TKA with whole-body EOS X-ray (EOS imaging, SA, Paris, France) imaging serial (preoperative and 3-month and 1-year postoperative) between February 2018 and September 2018. The exclusion criteria were secondary knee osteoarthritis (1 case), spinal fusion (12 cases), and hip surgery (2 cases). Consequently, 101 patients that underwent TKA, including both unilateral and bilateral TKA, were finally included with full serial assessment.

To evaluate the 3D knee and spinopelvic parameters, standing anterior-posterior (AP) and lateral whole-body EOS radiographs were used. INFINITT ver. 5.0.9.2 (INFINITT, Seoul, Korea), which could measure up to 2 decimal places automatically, was used for the radiological measurements. The differences between postoperative passive motion and weight-bearing posture in the knee joint were assessed using the differences between the range of motion and genu varum (GV) assessment using a joint angle indicator in the clinic and real measurements on standing AP and lateral EOS images. We identified the patients who demonstrated this difference by evaluating the spinopelvic parameters. This study was approved by Institutional Review Board. 

### 2.2. Changes in Coronal Spinopelvic Parameters after TKA

**Figure 1:**
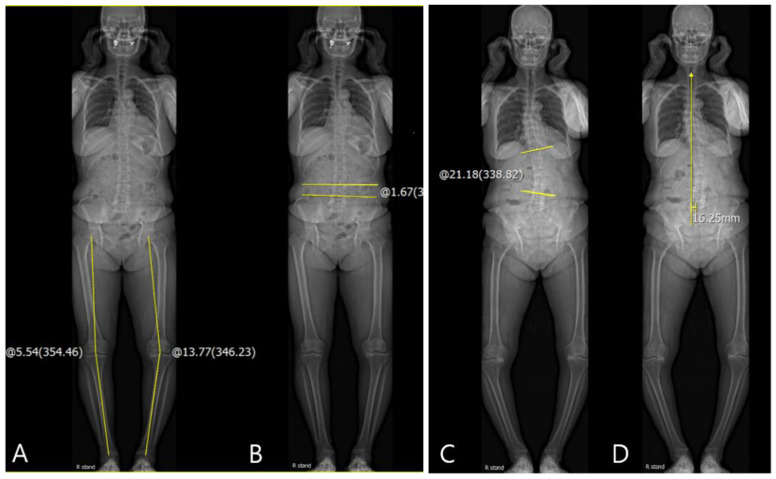
(**A**) Measurement of coronal HKA angle: coronal HKA_Rt-Lt_ = −8.23°; (**B**) measurement of pelvic obliquity: −1.67°; (**C**) measurement of the scoliosis angle: +21.18°; (**D**) measurement of coronal SVA: +16.25 mm.

For the evaluation of the coronal knee and spinopelvic parameters, coronal Hip-Knee-Ankle (HKA) angle, pelvic obliquity, scoliosis angle, and coronal C7 plumb line-sacrum distance (SVA) were evaluated on standing AP EOS images. For the HKA angle, a line was drawn from the center of the femoral head to the center of the knee, and a second line was drawn from the center of the knee to the center of the superior articular surface of the talus. The angle between the first and second lines was defined as the coronal HKA angle (Figure 1A) [3]. The coronal HKA parameter (HKA_Rt-Lt_) was defined as the difference between the coronal HKA in the right knee (HKA_Rt_) and that in the left knee (HKA_Lt_). Varus alignment was recorded as positive (+) and valgus alignment as negative (–). The coronal HKA parameter was positive if HKA_Rt_ > HKA_Lt_. The pelvic obliquity was measured as the angle between the reference horizontal line and the line connecting the uppermost borders of both iliac crests (Figure 1B) [12]. If the left iliac crest was higher than right iliac crest, it was recorded as positive (+). The scoliosis angle was measured as the angle between the most-tilted spinal vertebra in each curve. If the curve was to the left side and there was angular vertex on the right side, it was recorded as positive (+) (Figure 1C) [13]. The coronal SVA was measured as the distance between the vertical line from the midpoint of the C7 vertebral body to the mid-portion of the sacrum. If the coronal SVA was drawn to the right side of the sacrum, it was recorded as positive (+) (Figure 1D) [14]. The difference between preoperative values and those at 3 months and 1 year postoperatively were included in the serial assessments. Delta (Δ) was defined as the difference between the pre- and post-operative values. Correlations between the coronal ΔHKA parameters and Δspinopelvic parameters were also evaluated.

### 2.3. Changes in Sagittal Spinopelvic Parameters after TKA

**Figure 2:**
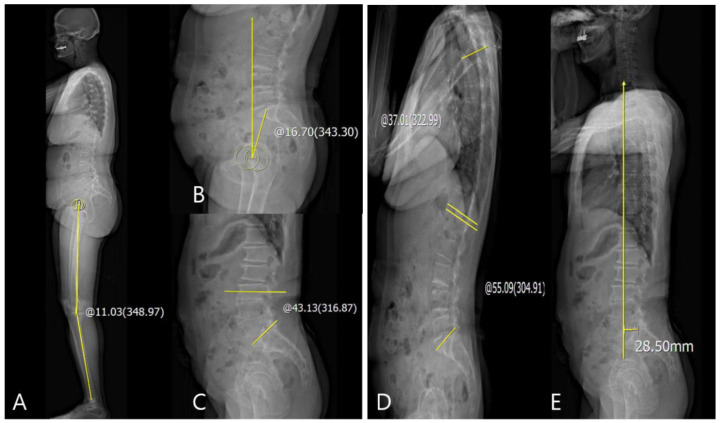
(**A**) Measurement of sagittal HKA angle: +11.03°; (**B**) measurement of PT: +16.70°; (**C**) measurement of SS: +43.13°; (**D**) measurement of TK and LL: +37.01° and +55.09°, respectively; (**E**) measurement of sagittal SVA: +28.50 mm.

For the evaluation of the sagittal knee and spinopelvic parameters, sagittal HKA angle, pelvic tilt (PT), sacral slope (SS), LL, thoracic kyphosis (TK), and sagittal SVA were evaluated on the standing lateral whole-body EOS radiographs. The sagittal HKA angle was measured as the angle between two lines: one joining the center of the bicoxofemoral head and the midpoint of each of the centers of knees and another joining the midpoint of each of centers of the knees and the midpoint of each of centers of the superior articular surface of talus. (Figure 2A) [3]. PT was measured as the angle between the line joining the center of the bicoxofemoral axis and the center of the S1 endplate and the reference vertical line (Figure 2B) [6]. SS was defined as the angle between the horizontal and the sacral plate (Figure 2C) [6]. LL was measured as Cobb’s angle between the cranial endplate of L1 and L5. TK was measured as the Cobb’s angle between the cranial endplate of T4 and T12 (Figure 2D) [6]. The sagittal SVA was measured as the distance between the plumb line from the center of the C7 to the posterior edge of the upper sacral endplate surface. If the sagittal SVA was drawn in front of the sacrum, it was recorded as positive (+) (Figure 2E) [15]. The difference between the preoperative values and those at postoperative 3 months and 1 year was included in the serial assessments. Delta (Δ) was defined as the difference between the pre- and post-operative values, and correlations between the sagittal ΔHKA parameters and Δspinopelvic parameters were evaluated.

### 2.4. Analysis of Difference between Passive Knee Motion and Weight-Bearing Knee

To identify the spinopelvic parameters that affect the difference between postoperative passive knee motion and weight-bearing knee posture, the differences between flexion contracture (FC) and GV assessments in the clinic and real measurements on standing AP and lateral EOS images at postoperative 1 year were evaluated, respectively. 

The difference in the coronal knee parameters was the difference between passive GV_Rt-Lt_ in the clinic and the weight-bearing coronal HKA_Rt-Lt_ angle on the radiographs. The difference in the sagittal knee parameters was the difference between passive FC_Rt-Lt_ in the clinic and the weight-bearing sagittal HKA_Rt-Lt_ angle on radiographs. The correlations between the differences in the coronal and sagittal knee parameters and spinopelvic parameters were evaluated.

### 2.5. Statistical Analysis

All data were analyzed using SPSS version 18.0 (SPSS Inc., Chicago, IL, USA). Data were described based on the means and standard deviations. Correlations between the knee parameters and other spinopelvic parameters in the serial assessments were analyzed with Pearson’s correlation and linear regression analysis. The inter- and intra-observer measurement reliabilities were assessed using the intra-class correlation coefficient. *p*-Values of <0.05 were considered significant for all tests.

## 3. Results

The average follow-up period was 12.37 months (range: 12–17 months). The inter- and intra-class correlation coefficients for the radiologic parameters were satisfactory with mean values of 0.91 and 0.87, respectively. The data of all parameters in both the coronal and sagittal planes are summarized in Table 1.

### 3.1. Changes in the Coronal and Sagittal Spinopelvic Parameters after TKA

**Figure 3:**
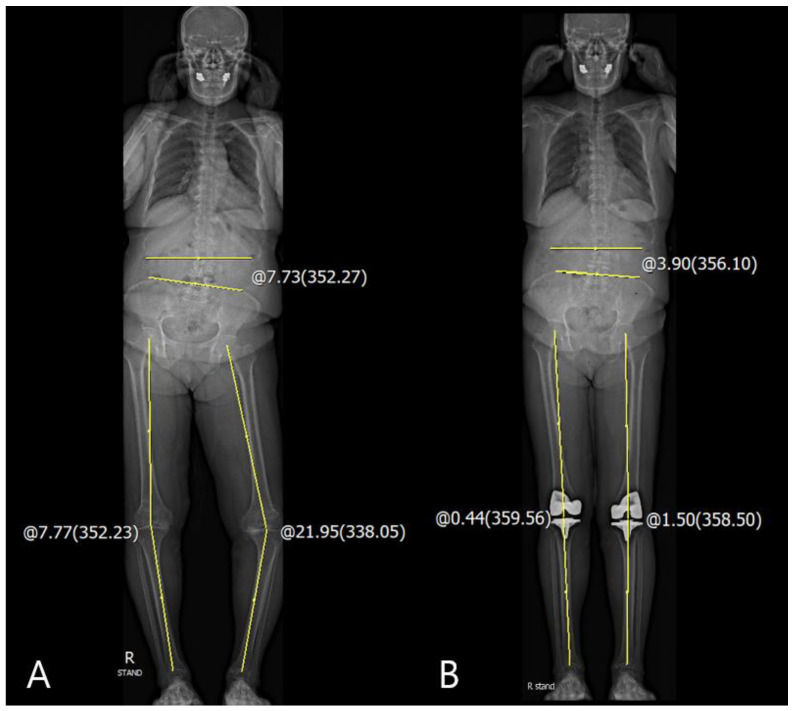
Corrected closed to neutral knee alignment affected the coronal pelvic obliquity. (**A**) Measurement of the preoperative coronal HKA angle: coronal HKA_Rt-Lt_ = −14.18° and pelvic obliquity: −7.73°; (**B**) measurement of the postoperative coronal HKA angle: coronal HKA_Rt-Lt_ = −1.06° and pelvic obliquity: −3.90°.

The correlations between the differences in the pre- and postoperative coronal HKA_Rt-Lt_ and other coronal spinopelvic parameters are summarized in Table 2. There were significant correlations between coronal Δpelvic obliquity and ΔHKA_Rt-Lt_ angle in preoperative, 3-month, and 1-year postoperative data (*p* < 0.001 and 0.005; Pearson correlation coefficient (PCC), 0.359 and 0.276, respectively). However, the scoliosis angle and coronal SVA were not correlated with the coronal ΔHKA_Rt-Lt_ angle. The linear regression analysis for the identification of cause–result relationship revealed that coronal ΔHKA significantly decreased pelvic obliquity (*p* < 0.001, ß = 0.085 and *p* = 0.005, ß = 0.065, respectively) (Table 3 and Figure 3).

**Figure 4:**
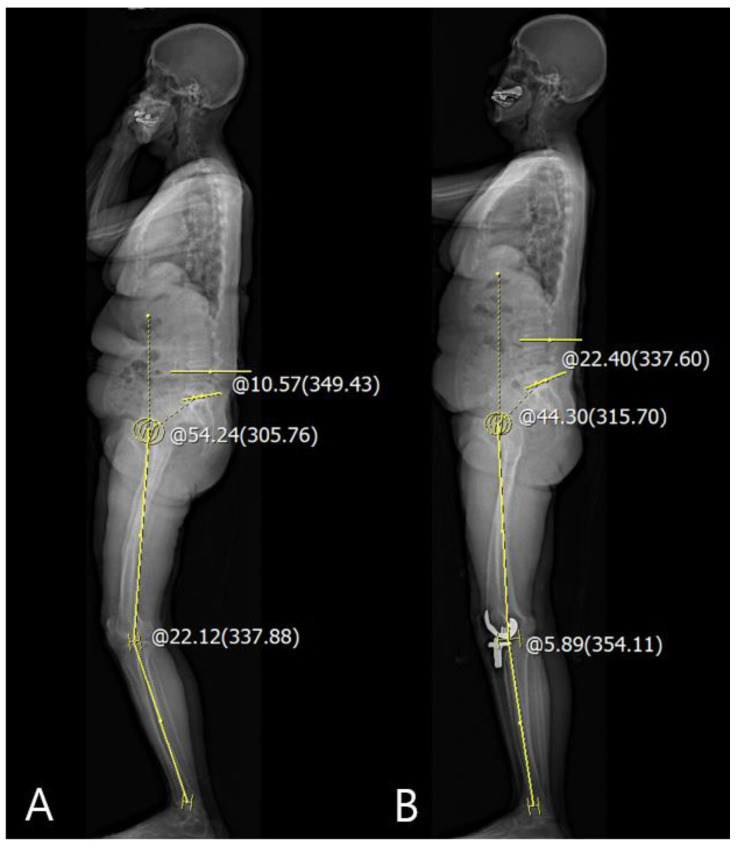
Corrected closed to neutral knee alignment decreased PT and increased SS. (**A**) Measurement of the preoperative sagittal HKA angle: sagittal HKA_Rt-Lt_ = +22.12° and preoperative PT: +54.24° and SS: +10.57°; (**B**) measurement of the postoperative sagittal HKA_Rt-Lt_ = +5.89° and PT: +44.30° and SS: +22.40°.

The correlations between the differences in the pre- and postoperative sagittal HKA_Rt-Lt_ and other sagittal spinopelvic parameters are summarized in Table 4. There were correlations between ΔPT and ΔSS as the sagittal pelvic parameters and sagittal ΔHKA_Rt-Lt_ angle in preoperative, 3-month, and 1-year postoperative data (PT: *p* < 0.001 and 0.045, SS: *p* = 0.002 and 0.001; PCC: 0.341 and 0.200, −0.306 and −0.322, respectively). However, TK, LL, and sagittal SVA as sagittal spinal parameters were not correlated with the sagittal ΔHKA_Rt-Lt_ angle. Linear regression analysis demonstrated that sagittal ΔHKA was significantly associated with ΔPT and ΔSS, inducing a decrease in PT and increase in SS from the preoperative to postoperative period (Table 3 and Figure 4).

### 3.2. Analysis of Difference between Passive Knee Motion and Weight-Bearing Knee 

**Figure 5:**
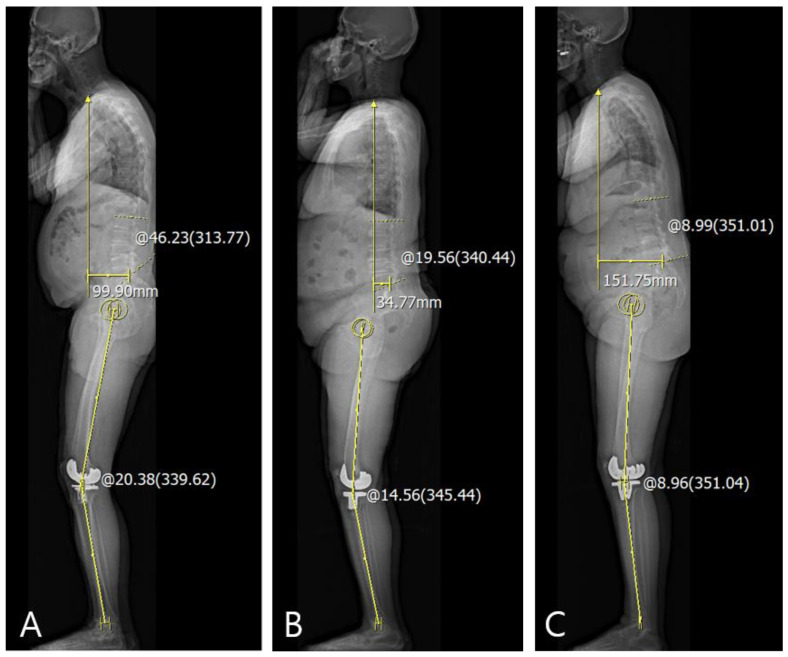
The difference between passive motion and weight-bearing of knee joint predominantly appeared if lumbar lordosis decreased or sagittal C7 plumb line was anteriorly located. (**A**) patient with anterior stooping because of anteriorly located C7 plumb line; (**B**) patient with decreased lumbar lordosis; (**C**) patient with combined anterior stooping and decreased lumbar lordosis.

The correlations between the differences between the clinical and radiological (GV or FC) data and other spinopelvic parameters at 1-year postoperatively are summarized in Table 5. There were correlations between the difference in the sagittal FC and sagittal spinal parameters, such as LL and sagittal SVA at 1-year postoperatively (*p* = 0.042 and *p* < 0.001, respectively; PCC= −0.209 and 0.412, respectively). However, none of the coronal spinopelvic parameters were correlated with the difference in the coronal GV. Linear regression analysis demonstrated that LL and sagittal SVA were both significantly associated with differences in sagittal FC (Table 6). These differences appeared predominantly if LL decreased and/or the sagittal C7 plumb line was located anteriorly (Figure 5).

## 4. Discussion

The principal findings of this study were as follows. First, following the changes in coronal and sagittal ΔHKA after TKA, the pelvic parameters demonstrated a significant compensation mechanism when compared to the spinal parameters. Particularly, a high correction of GV and FC after TKA—indicating an increase in ΔHKA in both planes—decreased the pelvic obliquity and pelvic tilting and increased the sacral slope. Second, LL and sagittal SVA were related to the difference in FC between clinically measured and actual weight-bearing radiograph data. The difference was predominantly observed in patients with decreased LL and/or anteriorly positioned sagittal C7 plumb line. 

The bi-planar low-dose EOS system has been used to develop a new modality for clinical alignment analysis [16]. Two perpendicular X-ray beams move vertically with the patient standing in the center of the scanning booth. The entire body, or a part of it, is scanned with simultaneous projections in two perpendicular planes without magnification. Although EOS is expensive and not so widely used, the radiation dose for the patient is substantially lower than that in conventional radiographs [16,17]. As part of the preoperative work-up for total hip arthroplasty, the EOS system provides information on the overall body alignment, especially the alignment of the lumbar spine and the pelvis, which is important before replacement surgeries [18,19]. However, as a part of TKA, the EOS system is rarely used to analyze the implant positioning after TKA and assess the prosthesis alignment after revision TKA [20,21].

Several previous studies have been published regarding the relationship between spinopelvic and lower limb alignment. Lee et al. [22] described that the correction of FC after TKA only changed SS. Another study reported that LL could be reduced if the patient had a fixed FC of the knee [23]. However, in our study, patients with corrected fixed FC had significantly decreased PT and increased SS in the sagittal plane. Additionally, patients who corrected the fixed GV demonstrated decreased pelvic obliquity. These findings imply that the changes in limb alignment affected the proximal mobile segment, such as the pelvis, rather than the fixed degenerative spine.

We also observed an interesting phenomenon. Some patients tended to stand or walk with knee flexion even if the fixed knee FC was corrected. However, they were able to extend their knee fully during passive knee motion. In this study, we were able to identify the possible cause of this interesting phenomenon, postulating that it may be related to reduced LL and/or forward stooping [24,25,26]. This implies that the axial and lower limb alignments interact to aid in balance, which could be an important problem because this flexible knee flexion can become a fixed FC with time. Additionally, TKA is performed in older patients, who they have weaker quadriceps muscle strength when compared with to younger patients, which can accelerate the progression of FC. 

The clinical relevance of this study is that evaluating the whole-body alignment in both planes can be useful in predicting the standing alignment after TKA. It can provide prognostic information for patients who are scheduled for TKA and surgeons can preoperatively explain the possible whole-body alignment and standing posture following the procedure. 

There are several limitations in this study. First, the clinical and radiological measurements of FC were performed differently. Therefore, the radiological value was comparatively larger than the clinical value. However, the radiologic measurement used in this study with an EOS image would be more appropriate for accurate quantitative analysis. Second, the follow-up period was short and the clinical results such as body mass index (BMI) are lacking because we only focused on the relationship between the axial and lower limb alignments after TKA. Third, this is a retrospective study, and the results have weak correlations, indicating possible selection bias. Fourth, changes in the apparent clinical symptoms, such as walking discomfort and lower back pain, were not included in this study 

## 5. Conclusions

The correction of lower limb alignment using TKA affected pelvic parameters dominantly; however, spinal alignment was little affected. Additionally, patients with anterior stooping or lumbar flat back demonstrated difference in extension between passive knee motion and standing. Therefore, rather than only focusing on changes in the knee alignment correction, knee surgeons should also evaluate the spinopelvic alignment before surgery to consider the prognosis of the standing and predict the possible changes in the whole-body alignment. This preoperative assessment may improve the prognosis of TKA.

## Figures and Tables

**Table 1:** Data of all the coronal and sagittal parameters.

Parameter	Preoperative	Postop 3 Months	Postop 1 Year
**Clinical parameters**			
FC (°) Rt	9.85 ± 6.26		0.24 ± 1.09
FC (°) Lt	10.19 ± 5.65		0.29 ± 1.19
GV (°) Rt	8.66 ± 6.04		0.49 ± 1.50
GV (°) Lt	8.76 ± 6.06		0.25 ± 1.09
**Coronal parameters**			
Coronal HKA angle (°) Rt	8.95 ± 6.08	2.82 ± 2.33	3.18 ± 2.45
Coronal HKA angle (°) Lt	9.08 ± 5.83	2.69 ± 2.78	2.91 ± 2.87
Scoliosis angle (°)	6.85 ± 5.18	6.56 ± 4.90	7.68 ± 9.63
Coronal SVA (mm)	11.64 ± 10.02	10.02 ± 7.66	2.25 ± 14.04
Pelvic obliquity (°)	1.81 ± 1.56	1.77 ± 1.42	−0.96 ± 2.10
**Sagittal parameters**			
Sagittal HKA angle (°)	10.57 ± 5.81	7.22 ± 5.71	5.52 ± 6.22
TK (°)	33.82 ± 10.43	33.07 ± 10.66	31.29 ± 11.03
LL (°)	48.42 ± 12.47	46.21 ± 11.47	46.62 ± 12.45
Sagittal SVA (mm)	15.80 ± 33.54	29.45 ± 36.99	26.01 ± 33.79
PT (°)	21.36 ± 8.73	18.87 ± 8.05	18.68 ± 8.07
SS (°)	34.70 ± 7.96	37.61 ± 8.39	35.73 ± 8.00

FC: Flexion contracture; GV: Genu varum; SVA: C7 plumb line sacrum distance; TK: Thoracic kyphosis; LL: Lumbar lordosis; PT; Pelvic tilitng; SS: Sacral slope; HKA: Hip-Knee-Ankle; Postop: postoperative; Rt: right; Lt: left. The values are presented as mean ± standard deviation.

**Table 2:** Correlation analysis between coronal ΔHKA_Rt-Lt_ angle and other parameters.

Parameter	Preoperative—Postop 3 Months	Preoperative—Postop 1 Year
**ΔScoliosis angle (°)**		
PCC	0.208	−0.003
Significant probability	0.057	0.977
**ΔSVA (mm)**		
PCC	0.013	0.115
Significant probability	0.894	0.253
**ΔPelvic obliquity (°)**		
PCC	0.359	0.276
Significant probability	<0.001	0.005

HKA: Hip-Knee-Ankle, Postop: postoperative, PCC: Pearson correlation coefficient. Δ: preoperative value—postoperative value, HKA_Rt-Lt_: HKA_Rt_—HKA_Lt.;_ SVA: C7 plumb line-sacrum distance.

**Table 3:** Subgroup analysis to identify the cause–result relationship using linear regression analysis.

Independent Variable	Dependent Variable	Preoperative—Postop 3 Months		Preoperative—Postop 1 Year	
		Regression Coefficient (*ß*)	*p*-Value	Regression Coefficient (*ß*)	*p*-Value
Coronal ΔHKA	ΔPelvic obliquity	0.085	<0.001 *	0.065	0.005 *
Sagittal ΔHKA	ΔPelvic tilting	0.354	<0.001 *	0.153	0.045 *
Sagittal ΔHKA	ΔSacral slope	−0.418	0.002 *	−0.348	0.001 *

Postop: postoperative; Δ: preoperative value—postoperative value. * *p*-value < 0.05.

**Table 4:** Correlation analysis between sagittal ΔHKA_Rt-Lt_ angle and other parameters.

Parameter	Preoperative—Postop 3 Months	Preoperative—Postop 1 Year
**ΔTK (°)**		
PCC	−0.010	−0.008
Significant probability	0.925	0.939
**ΔLLs (°)**		
PCC	−0.114	−0.026
Significant probability	0.254	0.793
**ΔSVA (mm)**		
PCC	0.046	0.015
Significant probability	0.650	0.881
**ΔPT (°)**		
PCC	0.341	0.200
Significant probability	<0.001	0.045
**ΔSS (°)**		
PCC	−0.306	−0.322
Significant probability	0.002	0.001

HKA: hip-knee-ankle, Postop: postoperative, TK: Thoracic kyphosis; LL: Lumbar lordosis; SVA: C7 plumb line-sacrum distance; PT: Pelvic tilting; SS: Sacral slope; PCC: Pearson correlation coefficient; Δ: preoperative value—postoperative value; HKA_Rt-Lt_: HKA_Rt_—HKA_Lt._

**Table 5:** Correlation analysis between difference in GV/FC and other parameters.

Parameter	Postop 1 Year
**Correlation between Difference in Coronal GV and Other Parameters**	
**Scoliosis angle (°)**	
PCC	0.098
Significant probability	0.328
**SVA (mm)**	
PCC	0.103
Significant probability	0.307
**Pelvic obliquity (°)**	
PCC	−0.036
Significant probability	0.722
**Correlation between Difference in Sagittal FC and Other Parameters**	
**TK (°)**	
PCC	−0.128
Significant probability	0.217
**LL (°)**	
PCC	−0.209
Significant probability	0.042
**SVA (mm)**	
PCC	0.412
Significant probability	<0.001
**PT (°)**	
PCC	0.197
Significant probability	0.056
**SS (°)**	
PCC	−0.054
Significant probability	0.607

Postop: postoperative; PCC: Pearson correlation coefficient. GV: Ggenu vara; FC: flexion contracture; TK: Thoracic kyphosis; LL: Lumbar lordosis; SVA: C7 plumb line-sacrum distance; PT: Pelvic tilting; SS: Sacral slope.

**Table 6:** Subgroup analysis using linear regression analysis.

Independent Variable	Dependent Variable	Regression Coefficient (*ß*)	*p*-Value
Lumbar lordosis	Difference in sagittal FC	−0.119	0.014 *
Sagittal C7 plumb line—sacrum distance	Difference in sagittal FC	0.087	<0.001 *

FC: flexion contracture. * *p*-value < 0.05.

## Abbreviations

TKA	Total knee arthroplasty
LL	Lumbar lordosis
3D	Three-dimensional
EOS	EOS^TM^ imaging system
GV	Genu varum
HKA	Hip-knee-ankle
SVA	C7 plumb line-sacrum distance
Δ	Delta
PT	Pelvic tilt
SS	Sacral slope
TK	Thoracic kyphosis
FC	Flexion contracture
PCC	Pearson correlation coefficient
